# Peer Review of “Exploring the Reasons for Low Cataract Surgery Uptake Among Patients Detected in a Community Outreach Program in Cameroon: Focused Ethnographic Mixed Methods Study”

**DOI:** 10.2196/38872

**Published:** 2022-06-09

**Authors:** Haleh Ayatollahi

**Affiliations:** 1 Iran University of Medical Sciences Tehran Iran

**Keywords:** ophthalmologic surgical procedures, access to health care, ophthalmology, patient-centered care, ethnography, health knowledge, attitudes, practice

*This is a peer-review report submitted for the paper “Exploring the Reasons for Low Cataract Surgery Uptake Among Patients Detected in a Community Outreach Program in Cameroon: Focused Ethnographic Mixed Methods Study.”*

## Round 1 Review

### Major Comments

1. It is better to choose keywords that are MeSH terms.
2. It is better to integrate all sections before *Methods* as an *Introduction* section.
3. How did the researchers develop the interview guide?
4. The trustworthiness of the results and validity and reliability need to be discussed separately for each research method.
5. More details should be added to the *Document Review* section.
6. How many participants took part in the focus groups?
7. The *Results* section needs to be expanded.
8. In the *Discussion* section, the summary of results does not need to be supported by the participant’s quotes.
9. The *Discussion* section needs to be revised to be more integrated.
10. Strengths and limitations of the study can be reported at the end of the discussion section.
11. Research implications can be reported before conclusions.

## Round 2 Review

### General Comments

I appreciate the authors [1] for their time and efforts to implement our suggestions. It seems that the manuscript has been improved; however, some issues are still remaining.

#### Specific Comments

##### Major Comments

1. The author response letter only includes the authors’ responses without mentioning the reviewers' comments. For some comments, they just said “done” and I have no idea what the comments were and what they exactly did. So, a complete response letter needs to be uploaded.
2. The *Discussion* section needs to be integrated to show an integrated *Discussion* for the whole research. In the current format, it seems fragmented.
3. Also, the subsections under the *Conclusions* section need to be moved to the end of the *Discussion* section or be integrated with other existing subheadings in this section.
